# Rates and relations of mitochondrial genome evolution across the Echinoidea, with special focus on the superfamily Odontophora

**DOI:** 10.1002/ece3.3042

**Published:** 2017-05-17

**Authors:** Áki Jarl Láruson

**Affiliations:** ^1^ Department of BiologyUniversity of Hawaiʻi at MānoaHonoluluHIUSA

**Keywords:** Echinoidea, gene evolution, mitochondrial genome, Odontophora, *Tripneustes gratilla*

## Abstract

In order to better characterize the placement of genus *Tripneustes*, as a representative of the Toxopneustidae family within the broader sea urchin mitochondrial (MT) phylogeny, the complete MT genome of *Tripneustes gratilla* was generated and compared with all published echinoid MT genomes currently available on NCBI GenBank. The MT genome phylogeny supports the existence of the superfamily Odontophora (consisting of the families Strongylocentrotidae, Echinometridae, and Toxopneustidae). A relaxed molecular‐clock time calibration suggests a split between the three key Odontophore MT lineages occurred during the late Eocene/Oligocene. Major global oceanographic changes have been inferred during this time frame, potentially driving species diversification through environmental selection pressures. To test for signatures of selection acting on the mitochondria, the historical rate of gene evolution of individual MT genes was assessed through a branch‐site comparison of nonsynonymous to synonymous substitution ratios (ω). Models of positive selection and neutral evolution, as compared via a likelihood ratio test, show no evidence of strong historical positive selection on mitochondrial genes at the genesis of the Odontophora. However, while pairwise ω comparison revealed signatures of strong negative selection, relatively elevated ω values were observed within the *Strongylocentrotus* genus.

## 1 INTRODUCTION

There are presently over 1,000 described species of sea urchins that collectively make up the class Echinoidea, the sea urchins, within the Phylum Echinodermata (WoRMS, 2017). Arising early on in the fossil record (Ordovician) as a distinct class, and lending themselves well to fossilization, echinoids have long been a hallmark of systematic research (Agassiz & Clark, 1907‐1917; Clark, 1912; Fell, 1974; Kroh & Smith, 2010; Littlewood & Smith, 1995; Smith, 1988; Smith & Savill, 2001). Following the publication of Theodore Mortensen's detailed alpha taxonomy of echinoids between 1928 and 1951, sea urchins became a tractable system to investigate questions of evolution and speciation (Mayr, 1954; Mortensen 1928–1954, Palumbi & Lessios, 2005).

Within the Echinoidea resides the ominously named Toxopneustidae family, whose members include the highly venomous “flower urchin,” *Toxopneustes pileolus* (Nakagawa et al., 1996), the much‐studied Caribbean “green sea urchin,” *Lytechinus variegatus* (Watts, McClintock, & Lawrence, 2013), and the Indo‐Pacific “collector urchin,” *Tripneustes gratilla* (Lawrence & Agatsuma, 2013), along with eight other recognized species (WoRMS, 2017). Though these species are well recognized and studied, their familial phylogeny is not as well established. Previous attempts at placement of the Toxopneustidae family within the greater sea urchin phylogenetic tree using morphology have suggested that the Toxopneustidae, Echinometridae, and the Strongylocentrotidae together form the superfamily Odontophora (Kroh & Smith, 2010). The complete MT genome of *T. gratilla*, reported here, represents the last genome needed in order to compare all MT family lineages within the proposed Odontophora superfamily.

Markers derived from MT genomes have long served as the molecular standard for species delineation, and population connectivity, as well as deeper evolutionary relationships (Avise et al., 1987; Ballard & Whitlock, 2004). Part of the appeal of the MT marker has been not only its ease of recovery during DNA extraction owing to the high copy numbers of MT genetic material per cell, but also the lack of recombination and a general assumption of freedom from strong positive selection (Grey, 1989). However, a growing number of studies have suggested natural selection on MT genes may not be as rare as previously assumed (Ballard & Whitlock, 2004; Bazin, Glémin, & Galtier, 2006; Doi, Suzuki, & Matsuura, 1999; Stojković et al., 2016).

Genetic variation of MT genes may be driven by thermal adaptation in ectothermic poikilotherms, such as sea urchins, as the thermal stability of transcribed proteins is critically important to function (Guderley & St‐Pierre, 2002; Hazel, 1995). Indeed, periods of great oceanographic change, including significant global ocean cooling, have been linked to species divergence as a result of climate‐driven selection pressures (Prothero & Berggren, 1992). Beyond thermal adaptation, cytonuclear incompatibility can also serve as a selection force on MT genes, which rely on nuclear molecular machinery. Cytonuclear incompatibility may arise through population divergence, as nuclear and MT genes evolve within a population, and can serve as a mechanism to restrict gene flow during secondary contact between populations (Burton & Barreto, 2012).

One approach to detect molecular signatures of selection across lineages is to quantify the proportion of nucleotide substitutions occurring at nonsynonymous codon sites compared to the proportion of synonymous substitutions, accounting for the degree of degeneracy at each codon site. This ratio of nonsynonymous to synonymous substitutions (d*N*/d*S* or ω) can serve as a highly conservative estimate of selection when comparing lineages. An assumption is made that the majority of synonymous substitutions are selectively neutral, while the occurrence of nonsynonymous substitutions are presumably selection driven. Values of ω close to 1 suggest neutrality, values much less than 1 suggest the action of purifying selection in removing amino acid substitutions, and values much greater than 1 pointing to positive selection on amino acid change (Kryazhimskiy & Plotkin, 2008; Nielsen & Yang, 2003). Extending this approach to inferred historical sequences of shared ancestors allows for an estimation of past instances of selection‐driven divergence. This study does just that by estimating ω values for each branch of a phylogenetic tree generated from complete MT genomes of 14 taxa across the Echinoidea, including 10 from the Odontophora. Two branch‐site tests of positive selection, a “strict” and “relaxed” variety, were performed on the branch giving rise to the Odontophora, in order to assess the influence of climate shifts driving lineage divergence through positive natural selection.

## 2 MATERIALS AND METHODS

Whole RNA was extracted from two adult *T. gratilla*, as well as from approximately 1,000 plutei‐stage *T. gratilla* larvae, using a Qiagen RNeasy extraction kit. All specimens were acquired from Oʻahu, Hawaiʻi. Poly A‐tail hybridization and cDNA generation were accomplished with an Illumina TruSeq kit and sequencing was performed on an Illumina MiSeq (300 cycle, single end) at the Hawaiʻi Institute of Marine Biology (HIMB) Core Genetics facility, Oʻahu, Hawaiʻi. Raw reads were filtered for adapter sequences using BBDuk in the BBMap package (Bushnell, 2015) and assembled in Trinity (v.3.1.1, Grabherr et al., 2011).

MT gene sequences were identified via annotation to the MT genome of the Strongylocentrotid *Hemicentrotus pulcherrimus* in Geneious (v.6). A draft MT genome for *T. gratilla* was generated by annotating the concatenated MT gene sequences at a 90% identity threshold to 12 echinoid MT genomes. All 13 coding DNA sequences (CDS), 22 tRNA, and two rRNA sequences were thus identified on the draft MT genome.

Using the draft MT genome, 40 pairs of novel primers were designed using the Owczarzy et al. (2004) salt correction formula, and thermodynamic parameters from SantaLucia (1998), as implemented in Primer3plus (http://www.primer3plus.com, Untergasser et al., 2012). A combination of regular and long‐range primers (used with NEB Long Range Taq 2x) were designed to generate overlapping products across the whole *T. gratilla* MT genome. An annealing temperature of 58°C was used for all thermocycler runs. DNA from a third adult *T. gratilla*, collected from Western Oʻahu, Hawaiʻi, was amplified using the novel primers in PCR, and resultant products were sequenced via Sanger sequencing on a 3730 ABI DNA Analyzer at the Advanced Studies in Genomics, Proteomics, and Bioinformatics (ASGPB), Oʻahu, Hawaiʻi. Overlap of resultant amplicons allowed for the inclusion of intergenic sequences and clarification of a few low‐quality areas from the draft MT genome to give a complete and high confidence MT genome sequence. The *T. gratilla* MT genome is available on NCBI GenBank (Accession number: KY268294).

In total, 12 complete MT genomes of echinoids were acquired from NCBI Genbank. This included *Arbacia lixula* (NC001770, De Giorgi, Martiradonna, Lanave, & Saccone, 1996), *Paracentrotus lividus* (NC001572, Cantatore, Roberti, Rainaldi, Gadaleta, & Saccone, 1989), *Loxechinus albus* (NC024177, Jung & Lee, 2015), *Sterechinus neumayeri* (NC020771, Dilly, Gaitn‐Espitia, & Hofmann, 2015), *Heliocidaris crassispina* (NC023774, Jung, Kim, & Lee, 2016), *Hemicentrotus pulcherrimus* (NC023771, Jung, Choi, Myoung, & Lee, 2014), *Mesocentrotus franciscanus* (NC024177, Gaitan‐Espitia & Hofmann, 2016), *Mesocentrotus nudus* (NC020771, Jung, Choi, Pae, & Lee, 2013), *Pseudocentrotus depressus* (NC023773), *Strongylocentrotus purpuratus* (NC001453, Jacobs, Elliott, Math, & Farquharson, 1988; Qureshi & Jacobs, 1993; Valverde, Marco, & Garesse, 1994), *S. droebachiensis* (NC009940), *S. pallidus* (NC009941), and *S. intermedius* (NC023772). Initially the MT genome of *Temnopleurus hardwickii* (NC026200, Fu, Liu, & Zeng, 2014), from the family Temnopleuridae, was included in the analysis. However, due to ambiguous phylogenetic signals across the *T. hardwickii* MT genome and a polytomous assignment of the species, it was dropped from the final analysis.

Full MT genome alignments were performed with MAUVE as implemented in Geneious v.6 (Darling, Mau, & Perna, 2010). Sequence length comparisons of the 15 MT genes were also performed in Geneious v.6. The two noncoding 12s‐rRNA and 16s‐rRNA sequences were aligned in T‐Coffee using the ribosomal folding algorithm implemented in the rcoffee mode (Notredame, Higgins, & Heringa, 2000; Wilm, Higgins, & Notredame, 2008). Comparative alignments of the rRNA sequences were executed with the Muscle (Edgar, 2004) and ClustalW (Thompson, Higgins, & Gibson, 1994) algorithms, as implemented in Geneious v.6, under default settings. The 13 coding DNA sequences (CDS) were aligned using the amino acid alignment algorithm in Geneious v.6, using the echinoderm MT codon table. Best fit nucleotide substitution models for the initial phylogenetic analysis for each of the 15 gene alignments were determined with JModeltest 2.1.7 (Darriba, Taboada, Doallo, & Posada, 2012).

From the 15 gene alignment dataset, a phylogenetic tree was generated with MrBayes 3.2.3, which was run for 5,000,000 steps; with a 1,250,000 step burn‐in (Huelsenbeck, Ronquist, Nielsen, & Bollback, 2001; Ronquist & Huelsenbeck, 2003), with *A. lixula* designated as an out group in order to root the tree. To confirm the robustness of the topology, a phylogenetic tree was also generated from the same data using RAxML v.8, which was run for 50,000 steps, with nonparametric bootstrapping enabled (Stamatakis, 2014). For the time calibration and gene evolution analysis a second Bayesian majority consensus tree was generated, under identical parameters as the prior tree, but including only the 13 CDS, not the 12S and 16S sequences. This CDS tree was generated to prevent confounding of codon substitution rate assessments with noncoding gene sequences.

Divergence calibration using the CDS majority consensus tree under a birth–death speciation process was performed in BEAST v1.8.7 (Drummond, Suchard, Xie, & Rambaut, 2012), with eight calibration points set. One log‐normally distributed fossil calibration point was set at the split of the Odontopohora from the Parechinidae (represented by *Loxechinus albus* and *Paracentrotus lividus*) and Echinidae (*Sterechinus neumayeri*), with an initial value of 42.5 mya (μ = 5, offset=40, σ = 1, Smith, 1988; Lee, 2003). All remaining node calibrations were normally distributed, and centered on splitting times derived from previous work on echinoid MT sequence evolution: *Sterechinus neumayeri* from the Parechinidae (μ = 29.5, σ = 4, Lee et al., 2004); *Loxechinus albus* from *Paracentrotus lividus* (μ = 25.5, σ = 3, Lee et al., 2004); root node of the Strongylocentrotidae (μ = 15.5, σ = 2, Lee, 2003); *Hemicentrotus pulcherrimus* from *Strongylocentrotus* (μ = 8.6, σ = 1, Lee, 2003); root node of *Mesocentrotus* (μ = 6.9, σ = 1.5, Lee, 2003); root node of *Strongylocentrotus* (μ = 5.6, σ = 1, Lee, 2003); and *S. droebachiensis* from *S. pallidus* (μ = 2.6, σ = 1, Lee, 2003). Partitioning schemes for models of molecular evolution for the fossil calibration run were determined with PartitionFinder v1.1.1 (Lanfear, Calcott, Ho, & Guindon, 2012). Marginal likelihood estimations using path sampling and stepping stone sampling were used for molecular clock models ranging from a strict to relaxed model and resulted in the selection of the relaxed log‐normal clock for the analysis (Baele et al., 2012). Calibrations were run for 100,000,000 generations and repeated five times, before being combined with logcombiner (Drummond et al., 2012). Proper Markov chain mixing was confirmed for both MrBayes and BEAST runs with Tracer 1.6 (Rambaut, Suchard, Xie, & Drummond, 2014). The resulting tree was visualized using the R packages phytools (Revell, 2012), PHYLOCH (Heibl, 2008), strap (Bell & Lloyd, 2014), and CODA (Plummer, Best, Cowles, & Vines, 2006).

A free rate model estimating the ratio of nonsynonymous to synonymous substitutions (ω) independently across every branch of the CDS tree was estimated with the codeml package within PAML (Yang, 2007; Zhang, Nielsen, & Yang, 2005), and pairwise ω were estimated with the Nei and Gojobori (1986) method. Three branch‐site models of ω variation along the Odontophore branch of the MT genome tree were then compared. The echinoderm‐specific MT codon table was specified for all calculations. Model MA fixes ω at 1 for every branch except for the specified branch leading to the Odontophora, where ω is assumed to be greater than 1. This model serves as the alternative to the two following null models. Model M1a similarly fixes ω at 1 for every branch except for the Odontophora branch, where ω is assumed to be ranging from 0 to 1; and MAnull fixes ω at 1 for every branch in the tree. Both MAnull and M1a were compared via a likelihood ratio test (LRT) to model MA. Comparing MA to MAnull is the “strict” branch‐site test for positive selection, as significance requires ω > 1 at some subset of codon sites along the Odontophore branch. The LRT of MA to M1a is the “relaxed” test of positive selection, as significance requires only that a subset of codon sites along the branch of interest display an elevated ω when compared to the same sites along the background branches, and can thus identify instances of relaxation of selective constraints as well as positive selection. Significance of two times the difference in likelihood (2Δℓ) was estimated on a 50:50 mix of point mass 0 and χ_1_^2^ distribution, with critical values of 2.71 for 5% and 5.41 for 1% significance levels (Self & Liang, 1987; Zhang et al., 2005).

## 3 RESULTS

The *Tripneustes gratilla* MT genome is 15,725 bp long, with a GC% of 40.3. This is just above the average MT genome length of 15,700.4 bp (s.d. 23.6) and mean GC% of 40.2. The largest MT genome so far reported is that of *P. depressus* (15,729 bp), while the smallest is that of *M. franciscanus* (15,649 bp). MT genome alignments in Mauve showed no gene rearrangements in any taxa. Comparative gene length summaries across the MT genomes revealed roughly four categories of nucleotide length variation across the 14 species. In order to compare length variance across genes of different length, the coefficient of variation was found by normalizing the standard deviation for each gene by the gene length. Category A (genes COX2, COX3, ND3, and ND4L) had no deviation in length across all sampled taxa; Cat. B (COX1, ND1, ND2, and ND4) averaged a coefficient of variation (CV) of 7.5 × 10^−4^; Cat. C (16S, ATP6, ATP8, and CYTB) had an average CV of 4.8 × 10^−3^; while Cat. D (12S, ND5, and ND6) had a value of 9.7 × 10^−3^. There was no significant correlation between gene length and CV (Pearson's *r* = 0.12). The categories did not strictly reflect the number of codon inserts or deletions across the samples. Values are summarized in Table 1 and visualized in Fig. S1.

**Table 1:** Average length and the coefficient of variation of the 15 MT genes across 14 echinoid taxa

Gene	Mean length	CV	CV category
ND4L	294	0	A
ND3	351	0	A
COX2	690	0	A
COX3	783	0	A
COX1	1,553.7857	0.000516	B
ND2	1,058.7857	0.000757	B
ND1	971.7857	0.000825	B
ND4	1,389	0.000885	B
162	1,539.2857	0.004198	C
ATP8	165.2143	0.004853	C
CYTB	1,144.5	0.004904	C
ATP6	691.0714	0.005278	C
ND5	1,914	0.009077	D
12S	893.2	0.009276	D
ND6	493.0714	0.010833	D

**Figure 1:**
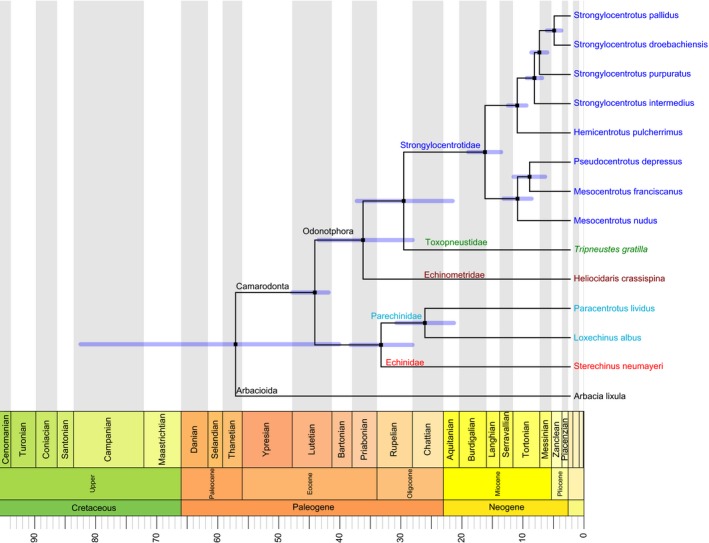
Bayesian tree showing mitochondrial genome relationships among the Echinoids. Posterior probabilities at all bifurcating nodes were 100%. Species are displayed to the right of the branch tips and color coded to their representative families. Blue error bars represent the 95% CI of the node height

Individual gene trees for 16S, ATP8, COX1, COX3, ND2, and ND3 recovered a monophyletic Odontophora superfamily. However, both ATP6 and CYTB failed to place *T. gratilla* in close association with Echinometridae and Strongylocentrodidae, while 12S, COX2, ND1, ND4, ND4L, ND5, and ND6 did not recover a grouping of Strongylocentrodidae with either Echinometridae or Toxopneustidae. The Bayesian consensus tree strongly supported the placement of *Tripneustes gratilla*, representative of the Toxopneustidae family, as the closest MT sister clade to the Strongylocentrotidae with a 100% posterior probability, as shown in Figure 1. Within the Strongylocentrotidae, the *Mesocentrotus* and *Pseudocentrotus* genera formed a unique clade, with *P. depressus* nesting within the *Mesocentrotus,* sister to *M. franciscanus. H. pulcherrimus* appeared as a sister taxa to the *Strongylocentrotus* genus. The four *Strongylocentrotus* species formed a clade with *S. pallidus* and *S. droebachiensis* as the most recent split, followed by *S. purpuratus*, and finally *S. intermedius*. Outside the Strongylocentrotidae and Toxopneustidae clade, the representative of the Echinometridae *Heliocidaris crassispina* branched off, completing the three family clusters identified as the superfamily Odontophora by Kroh and Smith (2010). Splitting from the Odontophora within the Camarodonta order are the families Echinidae (represented by *S. neumayeri*) and Parechinidae (represented by *P. lividus* and *L. albus*). *A. lixula*, the outgroup, displays the split between Order Arbacioida and the Camarodonta (Figure 1). A maximum likelihood tree generated with RAxML yielded an identical topology, so only the Bayesian tree is shown. The Bayesian consensus tree generated using only CDS (excluding 12S and 16S) also yielded an identical topology, and was used for ω estimation. Free rate estimation of ω for each coding gene across all branches suggested no strong signal at any one point in the CDS tree. Any elevated values of ω were found to be caused by synonymous substitution site proportions of zero, which inflated the effect of comparatively minute values of associated nonsynonymous substitution proportions. Although elevated nonsymonymous (dN) substitution rates were observed on the *S. purpuratus* branch, relative to all other branches within the Strongylocentrotidae. A one‐tailed *t* test, comparing the proportion of nonsynonymous substitutions averaged across all CDS (dN) of the *S. purpuratus* branch to the dN value averaged across the remaining seven branches of Strongylocentrodiae was significant at the 5% threshold with a Bonferroni correction (*n* = 182, *p = *1.117 × 10^−3^).

**Figure 2:**
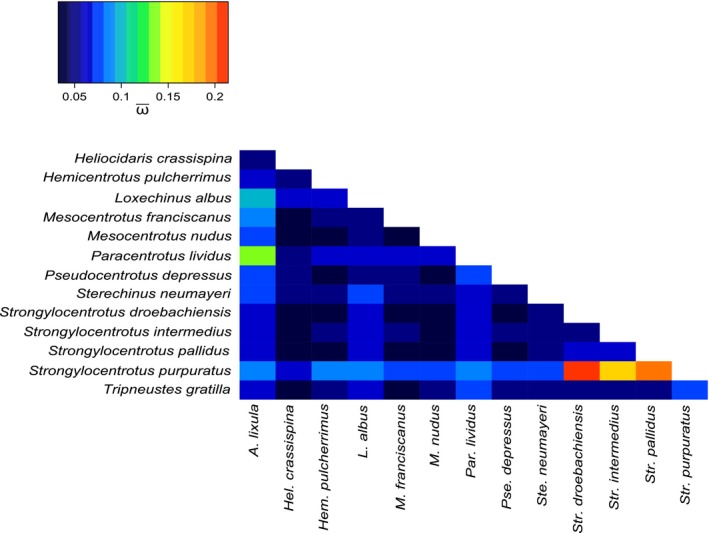
A heatmap depicting ω averaged across all 13 CDS, for each pairwise comparison. Lower values are depicted as cooler colors (blue), and higher values are presented as warmer colors (red). The largest values were consistently observed within genus *Strongylocentrotus*, specifically when *S. purpuratus* was compared to its congenerics *S. pallidus* and *S. intermedius*

Pairwise ω values were low across all genes, suggesting purifying selection, with one notable exception. ATP8 gave an ω value of 0.92 in the pairwise comparison between *P. lividus* and *A. lixula*, and values of 1.44, 0.94, and 1.06 in the pairwise comparisons between *S. purpuratus* and *S. droebachiensis*,* S. intermedius*, and *S. pallidus*, respectively Averaging across all gene pairwise comparisons, the highest relative ω values were consistently observed between *S. purpuratus* and its three congenerics. Average pairwise ω is summarized in Figure 2. Testing specifically the Odontophore branch, no CDS had significant results of positive selection according to the “strict” branch‐site test, with only three genes (ND1, ND5, and COX1) exhibiting differences greater than zero. Of those three, only ND1 showed a significant difference at the 5% level in the “relaxed” test for selection (Table 2).

**Table 2:** Results of the strict and relaxed branch‐site tests of positive selection

	ND1	ND5	COX1
Strict test			
2Δℓ	1.6998	1.0394	0.0157
*p*‐value	.0962	.1540	.4501
Relaxed test			
2Δℓ	4.2609	1.0394	0.2097
*p*‐value	.0195	.1540	.3235

Only difference values that were greater than zero are reported. p‐values are given for a 50:50 mix of point mass 0 and χ_1_^2^ distribution.

## 4 DISCUSSION

The placement of the Toxopneustidae family, represented here by the *Tripneustes gratilla* MT genome, as a closer sister group to the Strongylocentrotidae than the Echinometrid *Heliocidaris crassispina* contradicts the accepted species tree (Kroh & Smith, 2010). It must be specified that this relationship is based solely on MT sequences and is thus an insight into the evolutionary relationships of the Echinoid mitochondria, not a definitive depiction of the full evolutionary history of these species. Incomplete lineage sorting between the mitochondrial genome and the nuclear genome, or possibly lineage capture, could account for the contradictory signals of the MT and species tree in the sequence of splitting events at the cladogenesis of the Odontophora. The placement of *Pseudocentrotus depressus* as nested within the genus *Mesocentrotus* is consistent with previous MT gene analysis of the Strongylocentrotidae (Kober & Bernardi, 2013).

While comparing the individual gene phylogenies of the taxa considered here, it became clear that the choice in alignment method of ribosomal RNA 12S and 16S sequences had a significant impact on the phylogenetic signal of these two markers. Alignments using the built‐in algorithm of the Geneious software, as well as ClustalW, and Muscle alignments of the rRNA markers yielded trees for each marker that differed in topology from the CDS tree consensus. Yet, when the rRNA markers were aligned using the ribosomal alignment algorithm (rcoffee mode) in T‐Coffee, which uses ribosomal folding patterns as a cue, the 16S alignment resulted in an identical tree topology with the CDS consensus tree. Regardless of alignment method, the shorter 12S rRNA alignment was unable to resolve most relationships, showing primarily polytomies. 12S and 16S have been commonly used in many phylogenetic studies; however, the inability of either of these markers to recover even a resemblance of the consensus tree when aligned with commonly used nucleotide alignment algorithm could cast doubt on phylogenetic inferences derived solely from rRNA markers aligned via traditional means.

In contrast to the Smith et al. (2006), estimation of divergence times within the Class Echinoidea, which utilized fewer calibration points based on fossil dates of deep tree nodes, divergence estimates in this study relied on multiple calibrations of mostly shallow nodes. The calibration of the fully bifurcating tree indicates that the split between the three major Odontophores (Toxopneustidae, Strongylocentrotidae, and Echinometridae) occurred within a chronological range of the Eocene/Oligocene boundary. This period saw significant oceanographic changes due to a number of factors, such as the opening of south sea passage ways, and increased ice sheet coverage in the Arctic and Antarctic. A marked increase in atmospheric oxygen concentrations during this time contributed broadly to global cooling, with mean ocean surface temperatures estimated to have dropped by 4.4°C (Liu et al., 2009). This period gave rise not only to the Odontophora, but also to the splitting of the Parechinidae and Echinidae families; the Echinidae containing a prominent cold water sea urchin, the Antarctic *Sterechinus neumayeri*.

While modern psychrospheric (deep cold ocean) fauna are traced back to this period of large‐scale climate change (Prothero & Berggren, 1992), and the divergence of toothed and baileen whales appears to have been specifically driven by abiotic factors during this time (Steeman et al., 2009), no strong signature of selection was detected at the genesis of the Odontophora. Consistent with previous analysis, whole MT genome alignments show no gene rearrangements across the Echinoidea (Lee et al., 2004). The absence of regular recombination, generally assumed with mitochondria, can significantly inhibit the rates of fixation of advantageous mutants (Hill & Robertson, 1966). This could explain the lack of a strong positive selection‐driven signal. However, this does not discount the hypothesis that ecological and climatic shifts may have driven lineage diversification, but simply that MT genes were not predictably contributing. Nuclear genes, especially those involved in environmental stress response, could better indicate a pattern of abiotic selection pressure. Pespeni et al. (2013) showed that within *S. purpuratus* nuclear genes underlying some 40 functional classes of proteins undergo a shift in allele frequencies between populations reared at different levels of CO2 concentrations. As more nuclear markers and representative genomes become available for members of the echinoidea, a more definitive test of selection‐driven diversification within the Order will become feasible.

Mapping exclusively nonsynonymous (dN) proportions onto the CDS tree showed no single lineage with especially increased nonsynonymous substitution rates, save for one. The *S. purpuratus* branch across all MT genes consistently showed comparatively elevated nonsynonymous ratios, and the largest pairwise values of ω. As a model organism further study of the Strongylocentrotidae is warranted in order to determine whether the MT of *S. purpuratus* is indeed uniquely divergent from its congenerics due to selection (Sea Urchin Genome Sequencing Consortium 2006). However, consistent pairwise ω values much below one in most all taxa comparisons suggests that the major force affecting MT sequences is purifying, or negative, selection.

The generation of a complete MT genome of *Tripneustes gratilla*, the first for a member of Toxopneustidae, has allowed for the confirmation of the existence of the Odontophora superfamily. This underscores the fact that MT genomes can serve as a powerful analytical unit when estimating species relationships, especially in systems that may otherwise lack publicly available nuclear sequences for comparisons. It is the hope of the author that researchers generating NGS data will view the isolation, proper annotation, and dissemination of captured MT genome sequences to be a worthwhile endeavor.

## CONFLICT OF INTEREST

None declared.
