# Supplementary figures and images for: Rates and relations of mitochondrial genome evolution across the Echinoidea, with special focus on the superfamily Odontophora
Source: Ecol Evol. 2017 May 17;7(13):4543–51. doi: 10.1002/ece3.3042 (PMC5496550; doi:10.1002/ece3.3042)

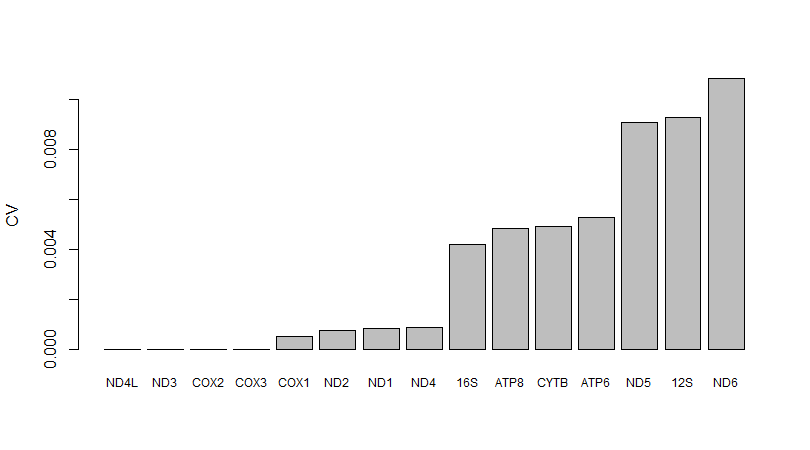

Supplement: Supplementary file 1 [file ECE3-7-4543-s001.png]
